# BatSLAM: Simultaneous Localization and Mapping Using Biomimetic Sonar

**DOI:** 10.1371/journal.pone.0054076

**Published:** 2013-01-24

**Authors:** Jan Steckel, Herbert Peremans

**Affiliations:** Active Perception Lab, Faculty of Applied Economics, University of Antwerp, Antwerp, Belgium; The Centre for Research and Technology, Greece

## Abstract

We propose to combine a biomimetic navigation model which solves a simultaneous localization and mapping task with a biomimetic sonar mounted on a mobile robot to address two related questions. First, can robotic sonar sensing lead to intelligent interactions with complex environments? Second, can we model sonar based spatial orientation and the construction of spatial maps by bats? To address these questions we adapt the mapping module of RatSLAM, a previously published navigation system based on computational models of the rodent hippocampus. We analyze the performance of the proposed robotic implementation operating in the real world. We conclude that the biomimetic navigation model operating on the information from the biomimetic sonar allows an autonomous agent to map unmodified (office) environments efficiently and consistently. Furthermore, these results also show that successful navigation does not require the readings of the biomimetic sonar to be interpreted in terms of individual objects/landmarks in the environment. We argue that the system has applications in robotics as well as in the field of biology as a simple, first order, model for sonar based spatial orientation and map building.

## Introduction

Bats have evolved sophisticated echolocation systems [1]–[3], which they use for a wide range of tasks such as spatial orientation, acquisition of food items, navigating complex and cluttered environments, etc. [4]–[6]. While researchers have already uncovered some of the principles that govern bat sonar, many open questions remain. One of those open questions, receiving more interest recently [7]–[10], concerns spatial orientation and the construction of spatial maps by bats using their sonar systems.

The problem of building spatial maps autonomously has been documented extensively in the robotics literature. Algorithms for Simultaneous Localization and Mapping (SLAM) combine information from proprioceptive sensors (shaft encoders, inertial systems, etc.) and exteroceptive sensors (vision sensor, laser range sensor, etc.) to estimate the position of the robot. At the same time a feature-based map of the environment is constructed, i.e. mapping objects onto parametric representations such as points, lines, circles, corners, etc [11]. Traditionally, this is done using probabilistic methods such as Kalman filters [12], Extended Kalman filters [13], or particle filters [14]. Most of these methods attempt to generate metric maps of the environment but methods for generating topological maps have also been investigated [15], [16]. While some of the original SLAM work used sonar sensors [17], [18], they have mostly been replaced by sensors providing richer and/or more fine-grained information such as optical sensors (camera's [19], laser range sensors [20]) or millimeter RADAR [21].

We argue [22] that the sparse and coarse grained environment descriptions resulting from standard robotic sonar sensors are a consequence of limiting in-air sonar technology to simple range sensors [23]. We propose that biosonar, on the other hand, is capable of supporting highly intelligent interactions with complex environments because it extracts much more information from the echoes. For example, the bat's sonar system recruits facial features such as the noseleaf [24] and outer ears [25], [26] to perform spectrospatial filtering on the emitted calls. Because of these interactions of the sound field with the bat's head morphology reflector location is encoded in a diverse set of monaural and binaural cues [7]. We have coined the term Echolocation Related Transfer Function (ERTF) for this spectrospatial filtering to emphasise that an active perception system can introduce such cues both during emission and reception. In [27] an information theoretic approach is described to quantify the amount of information (in bits) about the reflector location that can be extracted from the ERTF cues present in the echo signal. Based on this theory we have developed a biomimetic sonar system that is capable of localizing multiple reflectors in 3D using a single sonar emission [28].

Parallel to the traditional robotic simultaneous localization and mapping systems based on probabilistic methods, biologically inspired solutions have also been proposed. A highly successful example thereof is the RatSLAM system [29]–[31], which draws inspiration from the rat's hippocampal representation of space. Neuro-physiological experiments [32] show that the mammalian hippocampus and the bat's hippocampus in particular [9], [33] contain so called place cells. These cells are thought to encode the absolute position of the animal in its environment [34]. Loosely inspired by the rat's hippocampal mechanisms the RatSLAM system proposes a pose cell network, i.e. a continuous attractor network [35], [36], as a means to perform path integration. The pose estimate, i.e. robot position and orientation, resulting from this path integration is kept consistent by combining information from odometry, i.e. the measurement of self-motion, and vision. Different from traditional robotic SLAM techniques, RatSLAM does not require the data from the vision sensor to be segmented into a discrete set of object descriptions. Instead the visual data is used as a signature, a ‘fingerprint’, associated with a particular place in the environment. We propose to change RatSLAM into BatSLAM by replacing the vision sensor with a biomimetic sonar sensor.

The BatSLAM model for map building based on biomimetic sonar should be viewed as an experiment in synthetic psychology [37], [38]. The robotic implementation operating in the real world shows that the RatSLAM architecture combined with a biomimetic sonar system suffices for an autonomous agent to map office environments. In particular, it shows that consistent topological maps with semi-metric properties can be constructed using only motor commands and biomimetic sonar ‘fingerprints’. It also shows that if these sonar ‘fingerprints’ are sufficiently informative there is no need for further interpretation of them in terms of discrete objects positioned in the environment. As is often the case in biomimetic investigations, our goal is twofold. We want to show that biomimetic sonar sensing can support robots to interact intelligently with complex environments. In addition, we want to propose BatSLAM as a functional model of localization and map building by bats.

## Results

### Spatial sensitivity patterns of the biomimetic sonar system

**Figure 1:**
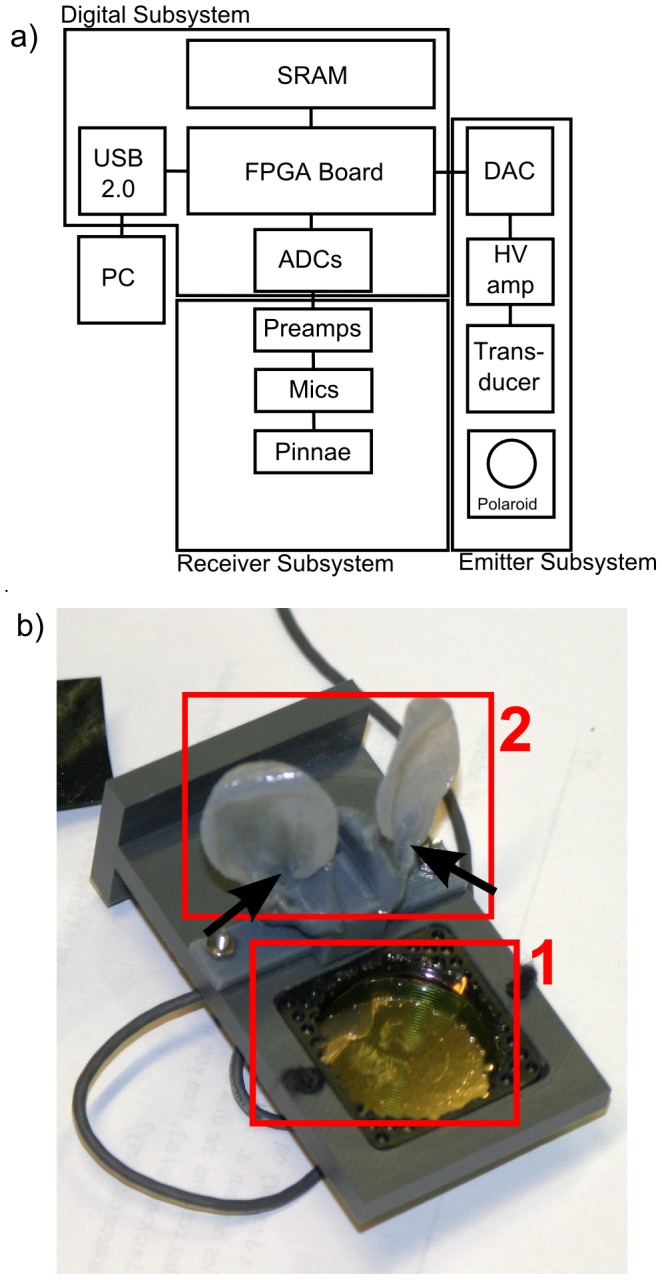
Detail of the used sonar system. The sonar system consists of an emitter and two receivers embedded in plastic replica of the bat's pinnae. a) Overview of the different functional blocks of the sonar system: digital subsystem with USB interface and 2 analog to digital converters; receiver subsystem containing 2 amplifiers and microphones; emitter subsystem containing digital to analog converter, high voltage amplifier and Polaroid transducer. b) Photograph of the constructed sonar head, showing the Polaroid transducer (1) and the microphones inserted in the pinna replica's (2). The position of the microphones is indicated by the black arrows.

**Figure 2:**
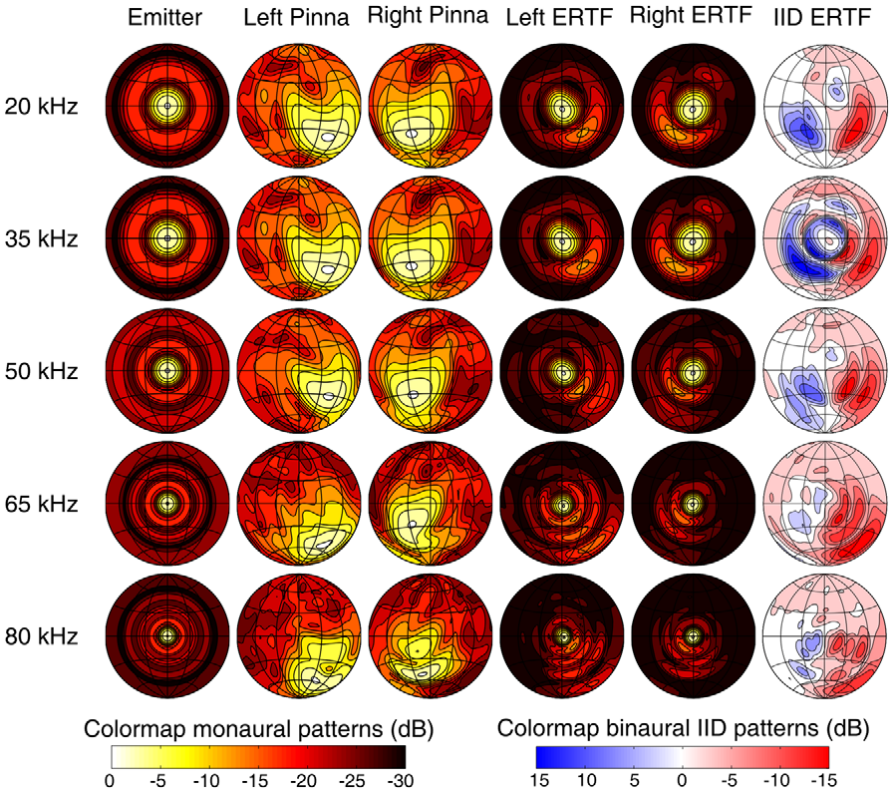
Sonar System directivity patterns. The emitter radiation patterns, the spatial sensitivity patterns for the left and the right ear, the ERTF for the left and right ear and the ERTF Interaural Intensity Difference patterns on a logarithmic scale. The grid lines are spaced 30°, the contour lines 3 dB. Note that the left and right pinna patterns are not exact mirror images, due to the asymmetric morphology of the scanned specimen.

For the exteroceptive sensory input to BatSLAM we propose to capture the echo signals through microphones inserted in replica's of real bat pinnae. See the Methods section for a detailed description of the biomimetic sonar system (Figure 1) used in the experiments. As known from spatial hearing investigations with bats [26], the spectrospatial sensitivity of a biosonar system is an important source of information regarding reflector location. Figure 2 illustrates the effect of the different components of the biomimetic sonar sensor on the overall spectrospatial sensitivity. It shows the simulated spatial sensitivity patterns for the emitter, the left (right) pinna and the left (right) Echolocation Related Transfer Function (ERTF). The ERTF is the multiplication of the emitter and receiver spatial sensitivity patterns as represented in the frequency domain. The binaural, interaural intensity difference, patterns are derived by subtracting right and left ERTFs.

These results show that informative spectrospatial cues are introduced both at emission and reception. Furthermore, comparing the monaural ERTF patterns with the interaural intensity difference patterns indicates that a binaural system can extract spatial information from a larger field of view. Finally, the ERTF results shown in figure 2 also indicate that the biomimetic sonar system is highly focused towards the frontal region. This can be understood by observing that the Polaroid transducer emits significant amounts of energy in a fairly small, forwardly directed, region only. As a consequence, the spectral features contained within the weaker echoes returning from more peripheral directions will be mostly masked by noise and thus uninformative. An information-theoretic analysis [27] along the lines of the one described in [39] can be used to quantify these statements.

### Mapping an office environment

Through the ERTF the sonar system maps the set of echoes from three dimensional reflector positions onto a two dimensional time-frequency representation, i.e. the cochleogram (see Methods). The smoothed cochleograms, denoted as local view templates, are fed into the otherwise unmodified RatSLAM algorithm together with the motor commands to generate a so-called experience map (see Methods). The experience map is a graph where the vertices represent unique places in the environment and the edges represent the robot displacements, as derived from odometry. While the experience map is essentially a topological map, the experiences are also assigned positions in experience space. These positions are initially based on information from the path integration module and subsequently refined upon loop closure (see Methods). As noted in [30] experience space is a manifold to the real world. This means that while it resembles Euclidean space on a local scale this need not be the case on a global scale.

#### Small-scale mapping accuracy

**Figure 3:**
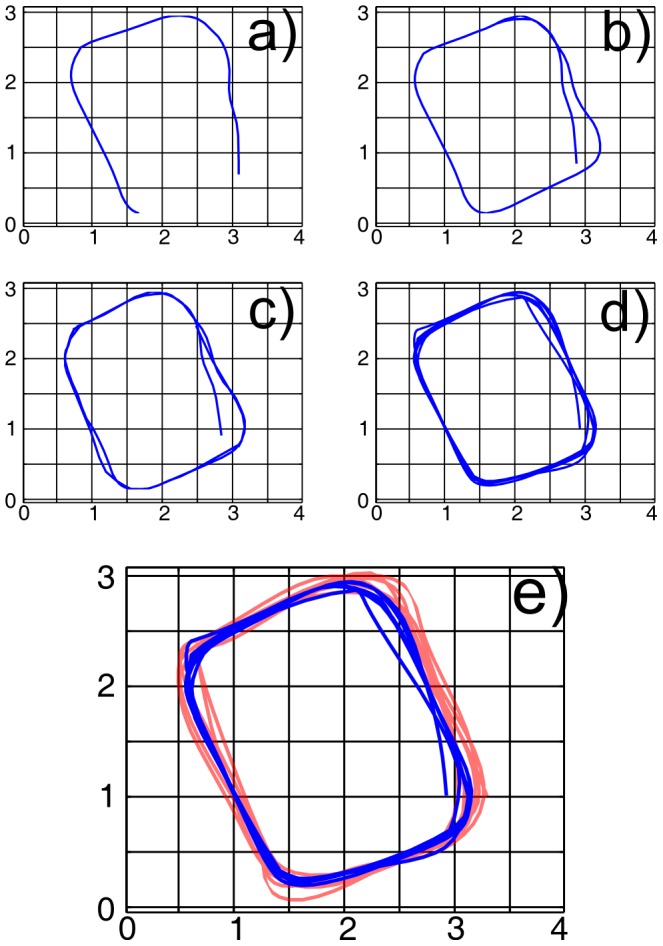
Small-Scale BatSLAM Mapping accuracy. Mapping result in a small scale environment using BatSLAM and a local positioning system as baseline. Subsets a–d) show intermediate trajectories generated by BatSLAM during the first few loops. In subset e) the red trace shows the robot trajectory measured with the Stargazer, the blue trace shows the robot trajectory in experience space as generated by BatSLAM.

In the first experiment, we quantify the local accuracy of the metric information in the experience map by mapping a controlled small-scale environment. In addition to the biomimetic sonar, a Hagisonic StarGazer [40] indoor localization system was mounted on the mobile robot. Using the indoor localization system, the real position of the robot was recorded for every sonar measurement collected along the test path. In total, 400 sonar measurements were recorded, with a mean displacement of 18 cm between two sonar measurements. The system generated 207 local view templates and 302 experience nodes. Figure 3 e) shows the Stargazer trajectory in red, and the robot trajectory estimated by the BatSLAM system in blue. The Pearson Correlation Coefficient [41] between the two trajectories (correlating the concatenated X and Y coordinates of both trajectories) is 0.994. This indicates that the true positions of the robot correspond very well with the assigned positions in experience space. Subsets a–b) show the BatSLAM trajectory during the first loop. Correct loop closure detection indicates recognition of previously visited places. Subsets c–d) show further convergence of the map over the next few loops. These results show that locally accurate metric representations can be built by BatSLAM.

#### Large-scale mapping

**Figure 4:**
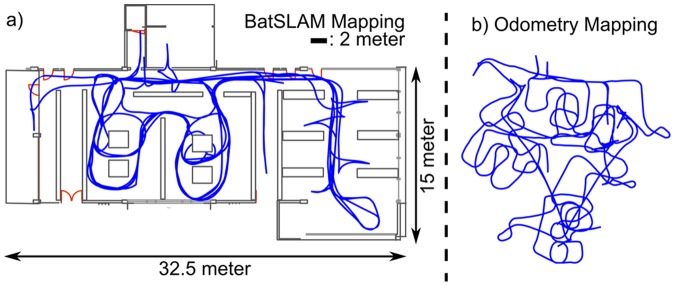
BatSLAM Mapping Result. Mapping result of an unmodified office environment using BatSLAM. a) The detailed experience map of the environment with the floor plan of the office building for reference. b) The map generated by applying a path integration algorithm directly to the motor commands.

To evaluate the large-scale mapping performance of BatSLAM we performed a second mapping experiment in a typical, unmodified, office environment. The biomimetic sonar system mounted on a mobile robot (height: 50 cm, pointing forward, elevation zero degree) was driven through the environment for approximately 45 minutes, while collecting sonar measurements. Consecutive measurements are separated by a mean displacement of 13.5 cm. The large scale of the environment precluded the use of the indoor localization system in this experiment. Figure 4 a) shows the experience map produced by BatSLAM. This result should be compared with Figure 4 b) showing the map resulting from applying naive path integration to the robot commands. We conclude that using only robot odometry does not, as expected, generate a useful map of the environment. However, the same odometry data combined with cochleogram data is turned into a consistent map by BatSLAM. A video detailing the gradual construction of the large scale map is uploaded as additional material.

Figure 4 a) also shows the actual floor plan of the office space superimposed, i.e. manually shifted and rotated but not scaled, on the experience map. From this result we conclude that even at this larger scale the metric properties of the positions in experience space still correspond quite accurately with those of the real world. This allows for metric path planning approaches using distance information to generate efficient paths in the real-world when applied to the positions in experience space.

#### Cochleograms as place descriptions

During the large-scale mapping experiment, the system collected 6000 sonar snapshots. Based on these 6000 sonar measurements, the BatSLAM system generated 4300 experiences in the experience map and stored 3300 local view templates. To allow a ‘fingerprint’ based mapping scheme like BatSLAM to construct accurate and stable maps these local view templates have to fulfill two conditions.

**Figure 5:**
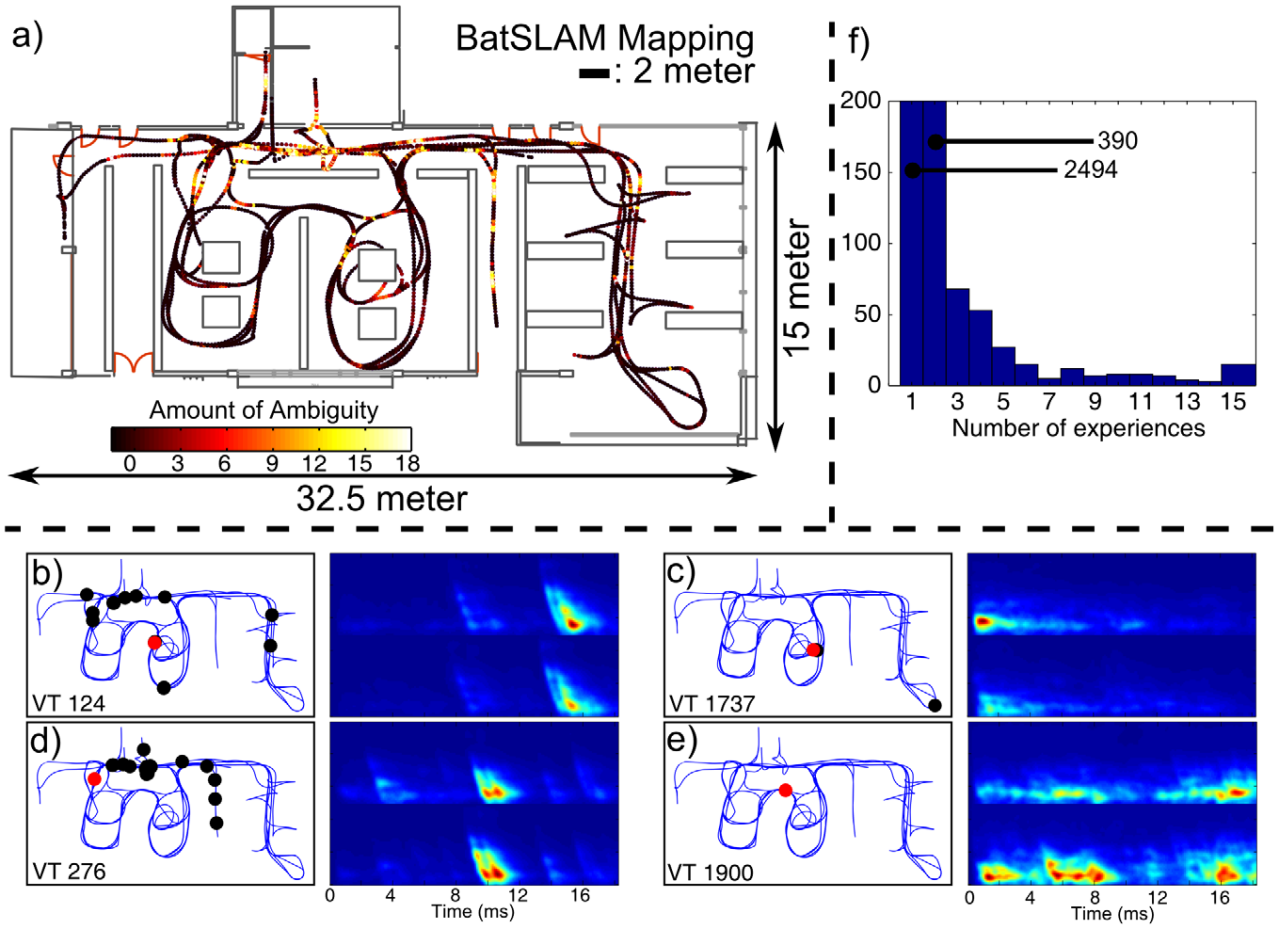
BatSLAM Mapping Ambiguities. The amount and the spatial distribution of ambiguity within the local view templates is shown to illustrate the performance of the BatSLAM system in typical office environments. a) The detailed experience map of the environment with the floor plan of the office building for reference. The color coding indicates the amount of ambiguity that exists for that particular position (see text). The red dots in b)–d) indicate the position where a particular local view template is encountered the first time. The black dots indicate where that same local view template is encountered again. The cochleograms show the actual local view template data. e) A local view template that is encountered only once.

First, the local view templates should be distinctive of particular places in the environment. As shown by the re-use histogram in figure 5 this condition is clearly fulfilled for the large majority of the cochleograms. Most collected cochleograms are associated with a unique place in the environment. A few cochleograms are linked with a much larger number of places. Indeed, as some places in the environment will generate similar sonar measurements, BatSLAM can associate different experiences, corresponding with different places in the real world, with the same local view template. This effect is illustrated in figure 5. In this figure the nodes in the experience map have been colored according to the number of times the associated local view template occurs anywhere else in the map. Zooming in on the actual cochleograms, figure 5b)–c) shows two non-distinctive local view templates and the different locations in the map where they re-occur. The first time a particular local view template is encountered is indicated by a red dot on the map, all subsequent occurrences are marked by black dots. The other two local view templates, figure 5d)–e), show that both very simple and very complex cochleograms can be highly distinctive of the places they correspond with. These results show that the office environment observed through the biomimetic sonar system gives rise to several regions of highly ambiguous ‘fingerprints’.

**Figure 6:**
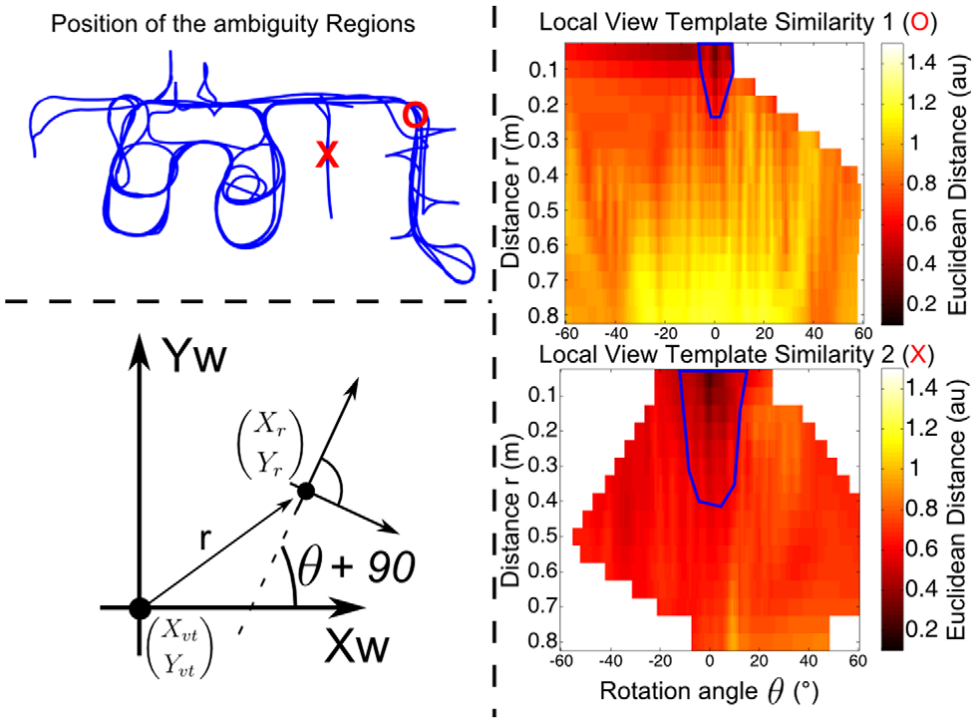
Local View Similarity Regions. The euclidean distances as a function of robot displacement for a typical position on the map (red circle), and for an ambiguous position on the map (red cross). The coordinate system used for the calculation is shown for clarity. The blue curves indicate the similarity regions, i.e. the outer boundary of the region wherein the local view templates are being recognized as similar to the original local view template.

In addition, the local view templates should have properties that change continuously in the neighborhood of the place they are distinctive of. This second condition is important for efficient loop-closure detection. It guarantees that similar robot poses, i.e. robot position and orientation, give rise to similar cochleograms thereby facilitating the recognition of already existing experiences, i.e. loop-closure. Figure 6 shows the spatial extent of the region associated with a particular local view template. First, the cochleogram corresponding with a particular position 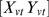 and orientation of the robot was recorded. The robot was subsequently moved to other neighboring positions (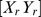) and orientations while collecting new cochleograms. Figure 6 shows the euclidean distance between the first local view template and the current one as a function of the robot displacement (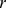, 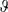). The first local view template is recorded at a typical location on the map, indicated by a red circle. For this position, the euclidean distance between the local view templates rises fairly quickly as the robot moves away from its original position. Note that with a mean displacement between consecutive sonar measurements of 13.5 cm approximately two sonar measurements fall within a typical similarity region assuming the robot heading does not change by more than 20 degrees in between measurements. Hence, the spatial sample rate of the sonar system seems well adjusted to guarantee loop-closure detection. The second local view template, indicated with a red cross in figure 6, originates from an ambiguous region, i.e. a region resulting in very similar cochleograms, in a long corridor. In this case, the euclidean distances between the local view templates change more slowly as the robot moves away from its original position. In particular, translations along the length of the corridor without rotations of the robot result in very similar local view templates. These results are to be expected from the structure of the corridor, i.e. two parallel walls with very little distinctive features along their length.

Large ambiguous regions could be a problem for BatSLAM as the robot can only rely on odometric information while moving within such an ambiguous region. However, from the results shown in figure 5 a), we conclude that the ambiguous regions have both a limited extent and a low frequency of occurrence. These results explain why BatSLAM can build robust and accurate maps of its environment relying on cochleograms that are not all uniquely distinctive.

## Discussion

From the results of the mapping experiments we conclude that BatSLAM is able to create consistent maps of large scale, unmodified, office environments. Furthermore, the constructed maps converge over time to fairly accurate metric maps supporting efficient, i.e. distance based, navigation through such environments. These results mirror the conclusions reached in [31] using vision sensing and large scale outdoor environments. By showing that sonar can support such intelligent interactions with complex environments BatSLAM makes possible a range of technical applications. Indeed, sonar, despite its limitations (specular reflections, sparse readings, etc. [22]) has distinct advantages over optical systems in low-visibility situations. Such situations occur in underwater environments but also in environments containing smoke or dust particles [42]. While traditional probabilistic SLAM systems with sonar sensors have been around for a long time [43], they typically require unrealistically large numbers of sonar readings to converge on usable maps. BatSLAM, by making more efficient use of the information contained in the sonar measurements, allows localization and navigation with realistic numbers of sonar readings [22] in a timescale appropriate for an autonomous robot. The mapping experiments also show that interpreting the environment in terms of individual objects is not necessary to generate stable and consistent maps from sonar data. As a direct consequence however, a task like find object X cannot be solved directly with this system. BatSLAM can bring the robot to a particular place if it is known that object X is near that place but the search for the object itself needs to be done through other means.

We do not make any claims about the biological accuracy of the details of the proposed implementation for bat environment mapping. However, we propose that BatSLAM, being both explicit and biologically plausible, is a useful first-order model of bat localization and mapping. Clearly, the information required, i.e. the steering commands and the output of the cochlear model [44], is available to the bat. Furthermore, regarding the components of the hippocampal core model, place cells [9] have also been found in the bat hippocampus. Hence, the only component for which there is no direct evidence is the experience map, i.e. a topological map of the environment with semi-metric properties. However, bats are known to be capable of middle-scale navigation defined in [4] as ‘the ability of bats to reach goals beyond the operating range of the echolocation system but within their home territory’. This capability requires a mechanism for building and maintaining a spatial representation of the home territory. Hence, indirect evidence for the use of a map is provided by the observation that many bats follow distinct and temporally stable flyways when flying between roosts and feeding grounds [45]. Specific experiments would have to be designed to distinguish between a purely topological representation as proposed in [4] and a semi-metric representation as proposed here. Maze experiments that block the originally learned path to a target and observe whether the alternative paths chosen by the bats are based on metric information or not could make this difference clear.

A necessary condition for an agent to follow a route or localize itself on a map of an environment is that the agent has access to unique descriptions of the places in that environment. In [4] it is suggested that these places are uniquely characterized by the spatial configuration of a set of landmarks that can be individually recognized. Different from this suggestion, BatSLAM generates such unique place descriptors by combining rough robot displacement estimates, i.e. motor commands, with often ambiguous cochleogram encoded views of the environment. We find indirect evidence for such a joint odometry-sonar based representation of the environment in the typical routes followed by bats during middle-scale navigation. The observed flight routes, i.e. fairly straight segments traversed at constant speed interspersed with discrete direction changes [4], [45], provide optimal conditions for odometry based position and orientation estimation. The spectrospatial filter of a binaural sonar maps a set of three dimensional reflector positions onto the corresponding cochleogram. For a realistic spectrospatial filter the mapping can be inverted to extract position information about individual objects in the environment. This process is straightforward for isolated echoes from a single object [28]. When echoes from multiple objects overlap the extraction of position information becomes ill-posed and needs to be regularized [46]. It is not known whether bats perform similar processing on incoming echoes to extract individual object position estimates. In the absence of more specific evidence we have opted not to decode cochleograms into object based environment descriptions. Instead, we use cochleograms as ‘fingerprints’ for particular locations in an environment. Hence, no mechanisms to identify and localize individual reflecting objects are included in BatSLAM. Information about the environment is only implicitly encoded by spectro-temporal features of the cochleogram representation ([27], [47]). The experiments described in [8] already show that bats can rely on sonar only for spatial orientation. The success of BatSLAM shows that sonar based localization and mapping in bats could possibly be explained without the need for assuming object identification and localization capabilities.

Extrapolating from the uniqueness of the complex cochleogram shown in figure 4e) we conjecture that a more panoramic view of the environment results in more stable place recognition and mapping. In that case, the ERTF results shown in figure 2 suggest that further improvements in performance can be expected from the use of an emitter with a broader beamwidth. Indeed, the widths of both measured [48] and simulated [24] emission beams indicate that bats have broader emission beams than the sonar sensor used here [49]. However, at least some bats reduce the width of their emission beam [48] depending on the context. Our analysis of the effects noseleaves have on the emission beams in broadband bats [24] points out two advantages in doing so. First, by focusing the energy into a smaller focal area, the bat will be able to detect weaker echoes from targets in this focal area than it would otherwise. Secondly, a more narrow emission beam enhances the difference in energy between peripheral echoes and echoes from the focal area. This way, the target echo in the focal region will be less distorted by clutter echoes from the periphery. Hence, narrow beamwidth emissions, by maximally excluding interference from other object echoes, seem more appropriate when target centered tasks are performed. It would be interesting to compare the beamwidths of bats in the wild when traveling along flyways as opposed to bats localizing a landing site. The latter is defined in [4] as a small-scale navigation task, i.e. ‘a task in which the target of interest is within the perceptual range of the bat's echolocation system’. The reasoning above would predict broader beamwidths for middle-scale navigation tasks than for small-scale navigation tasks.

Many simplifications are involved in BatSLAM: 2D paths instead of 3D paths through the environment, an office environment instead of an outdoor environment, functional models of hippocampal structures and of the bat's sonar system instead of detailed copies. Nevertheless, we would like to argue that its success makes this model both a robust and easy to implement SLAM system to be used in real-life robotic applications as well as a useful, first order, computational model of the map building processes supporting middle-scale navigation in bats.

## Methods

**Figure 7:**
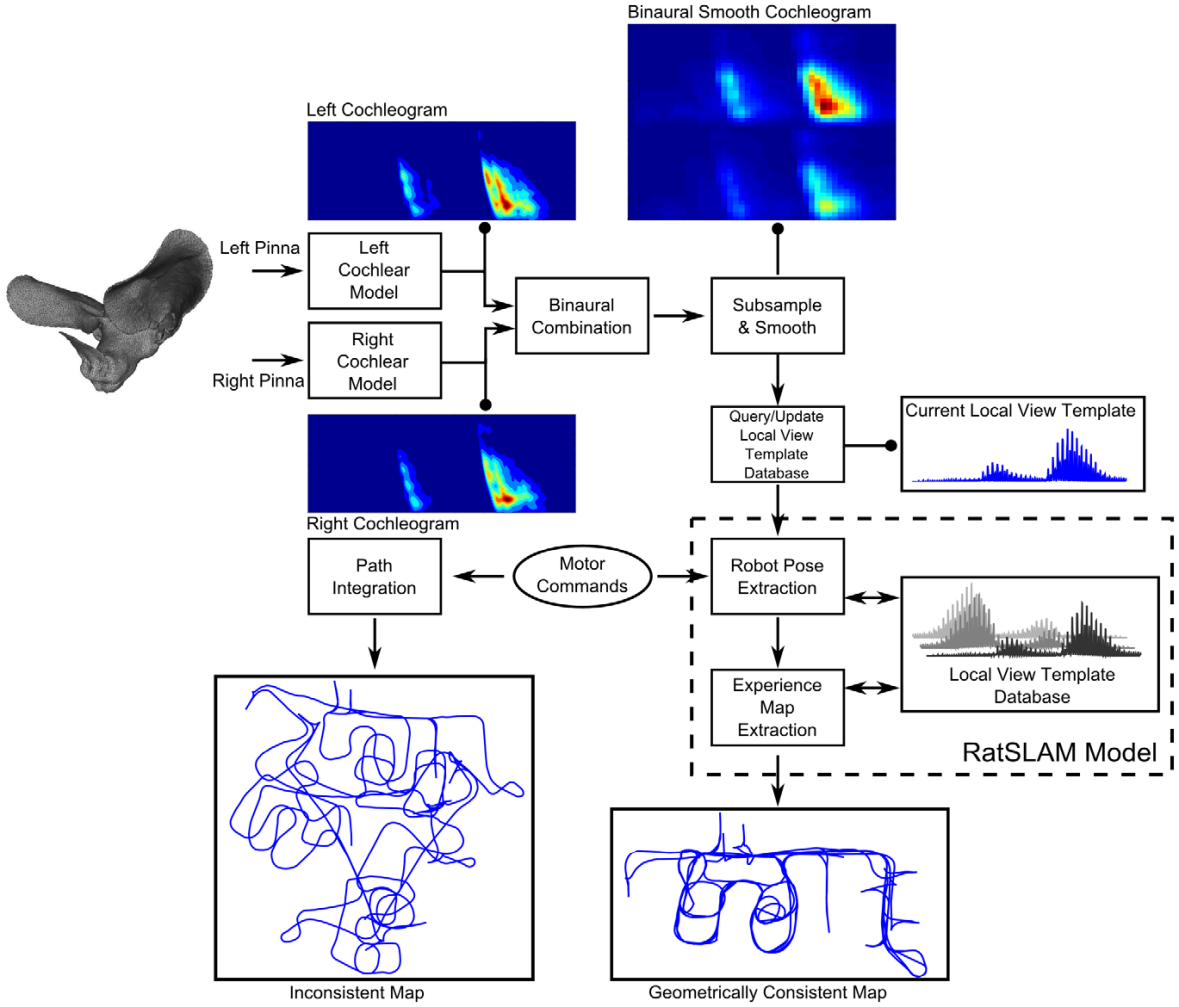
BatSLAM model processing steps. After signal reception by microphones inserted in the ear canals of plastic pinna replica's, the signals are processed by a functional model of a mammalian cochlea. The resulting cochleograms are smoothed and subsampled before being passed to the hippocampal core model as local view templates. The model constructs a database of local view templates. The similarity between the current local view template and the ones in the database determines the activity pattern over the view cell network. The pose cell network combines motor commands (proprioception) and cochleograms (exteroception) to produce an estimate of the current robot pose (position and orientation). An experience map is built from experience nodes representing unique combinations of the activity state of the pose cell network and that of the view cell network and linked by transitions representing robot displacements. The experience map (bottom right) is a robust and stable topological map of the environment with semi-metric properties. If only proprioception and path integration is used without environmental information from the sonar sensor, no consistent map can be constructed (bottom left).

This section provides details on the computational model implemented in the BatSLAM system. Figure 7 shows an overview of the performed operations. After signal reception by the right and left microphones inserted in the plastic pinnae, the signals are passed through a functional model of a mammalian cochlea. The resulting time-frequency representations of the echoes are denoted by cochleograms. These cochleograms are smoothed and subsampled before being passed on to the hippocampal core model ( = RatSLAM) as local view templates. This part of the model constructs a database of local view templates and generates an experience map by fusing information from the motor commands with the sonar data. After convergence, the output, i.e. the experience map, represents a robust and stable topological map of the environment with semi-metric properties. Note that the map contains only the trajectory that the robot has traversed throughout the experiment. The map does not contain a representation of the individual objects in the environment that generated the echoes.

### Biomimetic Sonar System

We start with an overview of the biomimetic sonar system and the signal processing applied to the incoming echo signals. For a detailed description and analysis of its performance the reader is referred to [28].

#### Sonar hardware

The different functional blocks that make up the custom-made sonar system are depicted in figure 1. The system consists of an Altera Cyclone I Field Programmable Gate Array, which connects to a computer via an USB 2.0 interface, allowing a maximum data transfer speed of 25MByte/sec. A MEX Matlab [50] interface gathers the data from the USB bus and presents it to the processing module. The emission subsystem consists of a 12 bit digital to analog converter with a sampling rate of 250 kS/sec connected to a high-voltage amplifier which in turn drives a single Polaroid transducer [23]. The receiver subsystem consists of 2 condenser microphones (Knowles FG-23329 [51]) with a diameter of 2.54 mm, inserted in the ear canal of plastic replica's of the outer ears of *Micronycteris microtis* scaled by a factor of 1.5 with respect to their true dimensions. Each microphone signal is amplified using a 6th order Butterworth anti-alias active filter with cutoff frequency at 150 kHz. Finally, the right and left microphone signals are digitized using 12bit analog to digital converters running at 500 kS/sec. The pick-up of the sonar emission by the receivers is removed by selecting the part of the microphone signal after the emission has stopped.

For the emitted signal we chose a hyperbolic chirp, i.e. a sinusoidal signal of 3 ms duration whose instantaneous frequency is hyperbolically swept from an upper frequency of 100 kHz down to a lower frequency of 20 kHz (the original frequency range of the bat spans from 50 kHz to 150 kHz [24]). The plastic pinnae are scaled by a factor of 1.5. This maps the interactions of the soundfield with the morphology of the outer ears from the original (higher) frequency range used by *Micronycteris microtis* [24] to the lower operational frequency range of the sonar hardware. The amplitude is modulated by a hamming window to minimize transient effects in the transducers and analog filters. High accuracy (0.1 mm) shape models of the *Micronycteris microtis* outer ears were obtained by micro-CT imaging [52] and replicated using 3D printing techniques.

#### Signal processing

Eqs. (1, 2) describe the echo filtering process resulting in the left (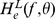) and right ear filter 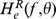


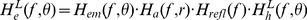
(1)

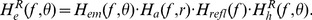
(2)

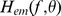
 denotes the filter associated with the emission process and 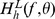, 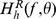 the left and right receiver filters for frequency 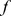 and direction 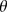. The angle 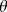 denotes the unique azimuth and elevation combination that specifies a reflector direction relative to the sonar system. The filtering due to sound propagation through air is taken into account by the factor 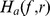. This factor includes frequency independent attenuation due to spherical spreading, frequency dependent absorption and the propagation delay introduced by the finite speed of sound [53]. Lastly, the filtering due to the interaction of the sound field with the shape of the reflector is denoted by 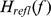. In general, this filter can change very rapidly depending on the pose of the reflector relative to the direction of the incident sound field ([27], [28]).

As all the signal operations are linear, the ERTF, which is the combination of the emission and the reception spatial sensitivity patterns can be written as
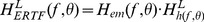
(3)

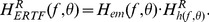
(4)Hence, in the presence of multiple reflectors, the received signals at the input of the cochleas for the left (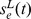) and the right ear (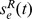) can be written as
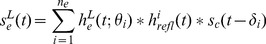
(5)

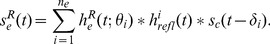
(6)In this expression 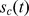 denotes the emitted call, 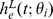 and 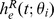 the impulse responses of the ERTF filters for the left and right ears respectively, 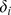 the delay introduced by the i-th reflector, 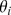 the direction of the i-th reflector relative to the sonar system, 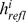 the filtering due to the i-th reflector, and 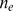 the total number of ensonified reflectors in the environment.

In order to analyze the features of these binaural echo signals in a biologically relevant time-frequency representation, we model the processing performed by the bat's cochlea. The operation of the cochlea is approximated using a bank of gammatone bandpass filters, followed by half-wave rectification, compression and a lowpass filter. The gammatone response 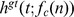 for the n-th frequency channel with center frequency 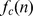 and bandwidth 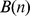, can be written as [54]

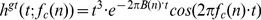
(7)Half-wave rectification 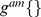 followed by a 1st order lowpass filter 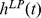 with a cutoff frequency of 200 Hz is applied to the outputs of the gammatone filters 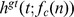. The cochleogram representations 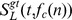 and 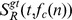 for the left and right ear can then be written as
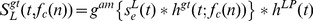
(8)

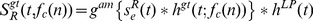
(9)with the operator 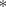 denoting time-domain convolution. As a last step, a logarithmic compression on the output data is performed, to obtain the cochlear representation of the input data.

### Sonar based local view templates

In BatSLAM, the cochlear representation of the input data is used to construct local view templates. For the biomimetic sonar system to replace the vision sensor in the original RatSLAM system, special care in the preparation of these sonar based local view templates has to be taken. First, the monaural cochleogram is subsampled in time to make the system more robust for small variations in robot position. Next, it is smoothed with a Gaussian filter to further increase the system's invariance to small position differences. Finally, the two smoothed and subsampled monaural cochleograms are concatenated to form a single binaural cochleogram 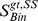


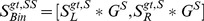
(10)with 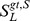 the left subsampled cochleogram, 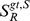 the right subsampled cochleogram, and 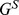 the Gaussian smoothing filter. As the intensity differences between the left and the right ear are very informative [47], the monaural cochleograms are not normalized. Figure 2 shows the interaural intensity difference patterns for the robotic setup, revealing that there is much spatial information encoded by the differences between the left and the right echo signals. However, for the system to be robust to small variations in overall echo strength (due to emission strength fluctuations, medium conditions, position and orientation errors), the binaural, smoothed cochleogram 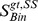 is normalized to have an energy content of one. This yields the normalized binaural smoothed cochleogram 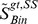


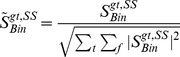
(11)We propose to use this version of the cochleogram as the current local view template 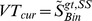. For every new sonar measurement the corresponding local view template 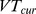 is tested to check whether it has been experienced before or not. This test is performed by calculating the euclidean distance 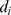 between 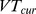 and all the stored local view templates in the database
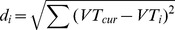
(12)with 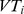 denoting the i-th template in the database. If the minimal euclidean distance is below a certain threshold 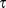, the new measurement is considered to match with the previous local view template 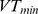 corresponding with this minimal distance. Through the local view cell associated with 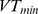 energy/activity is injected into the corresponding region of the pose cell network. The threshold 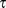 is calculated adaptively using a scaled version of the RMS value of the ensemble of all euclidean distances 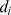


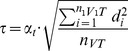
(13)with 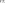 the number of local view templates in the database and 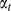 a scaling factor to tune the system. In our experiments, 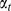 was set to 0.5. If the minimal euclidean distance remains above the threshold 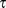, the scene is considered as new. In this case, 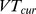 is added to the database and a new local view cell associated with 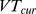 is added to the local view cell network.

### Spatial representation

**Figure 8:**
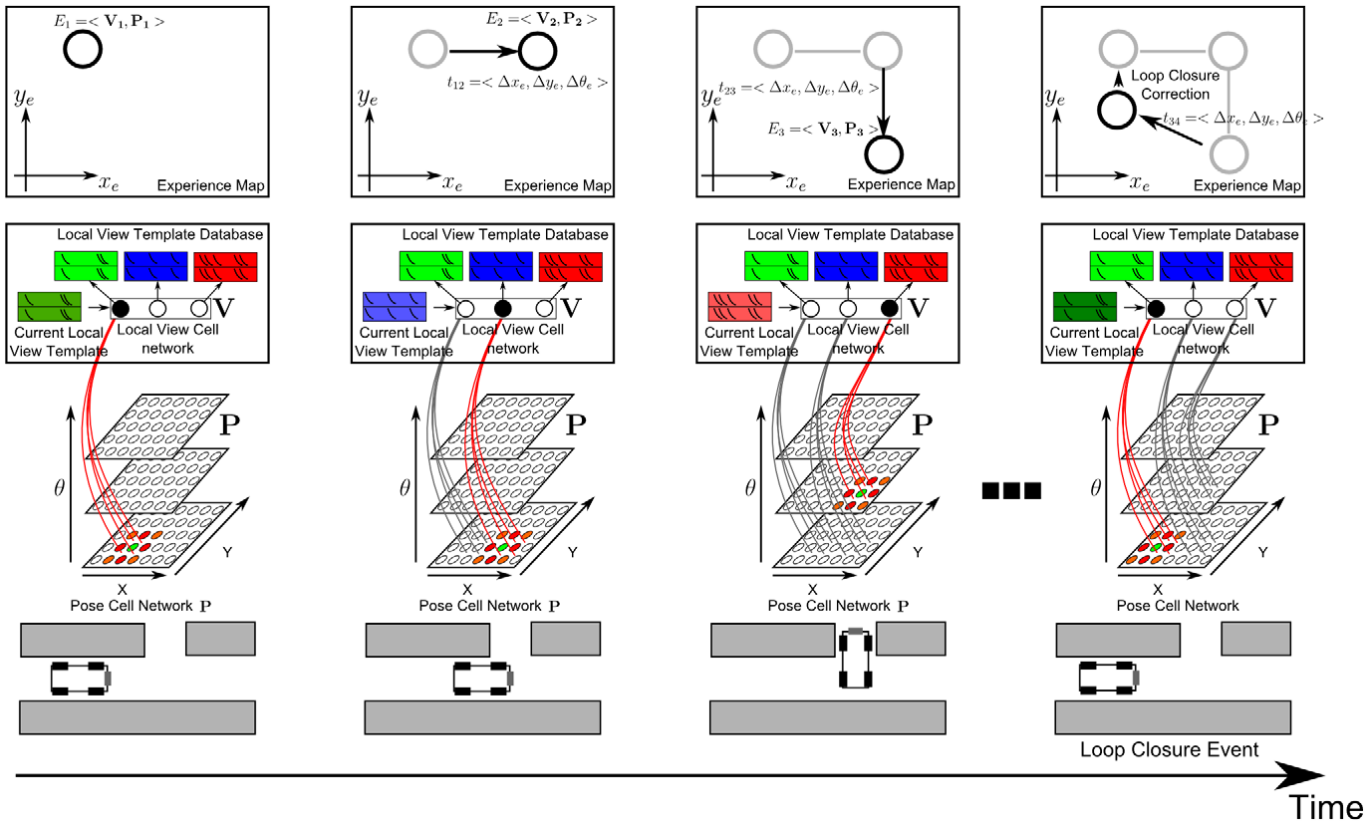
BatSLAM Core System. Overview of the core system that makes up the BatSLAM mapping module. Pose cell networks interact with local view cell networks to generate robust position estimates of a mobile agent. Sensor data extracted from the sonar system (indicated by Current Local View Templates) are compared to a database of previously encountered templates. If a template is recognized, the local view cell becomes active, correcting the position estimate in the pose cell network.

At the core of the BatSLAM system (see figure 8) [30] resides a functional model of the mammalian hippocampus. Cells that fire when the agent (robot/animal) is in a particular state ( = pose), characterized by the agent's position and orientation, are arranged in a three-dimensional grid (X, Y and 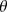 axis) to form a Continuous Attractor Network (CAN) called the pose cell network. Information about the robot's ego motion is incorporated by shifting activity packets within this network along the appropriate axes. In the implementation presented here, the commands sent to the motor subsystem of the robot, i.e. linear and rotational speed 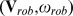, are used as first order approximations of the actual movements executed by the robot. The pose cell network combines this odometric information with information about the robot's environment through its interaction with the local view cell network. When the sensor system recognizes a particular scene, the current local view template matches a template from the database. As a consequence, the local view cell corresponding with the database template injects activity into the pose cell network. The injected activity activates mostly the pose cells, i.e. (X, Y and 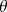)-regions, where the scene (local view template) is most strongly associated with. The links carrying the activity from the local view cells into the pose cell network are strengthened using Hebbian learning (see [29] for details). The dynamical properties of the CAN system assure that only a single activation peak can be maintained in the network thereby integrating the information from both the proprioceptive and the exteroceptive sensors into a unique robot pose estimate.

To make mapping of large areas possible without requiring equally large pose cell networks, the pose cell network exhibits a wrap-around topology. Consequently, activity in the same pose cells can code for the robot's presence at different places in the real world. Hence, to guarantee unique correspondence between places in the real world and the robot's representation, the concept of an experience is introduced. Experiences represent places in the real world. They are defined as tuples constructed by the conjunction of the activity state of the pose-cell network 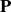 and the activity state of the local view cell network 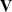. A new experience is created whenever the conjunction of current robot pose and current local view template differs more than a threshold value from all other experiences. Note that if the robot visits the same place in the real world but has a very different orientation a new experience will be created. In that case, neither the current robot pose, as it includes the orientation, nor the current local view template will match the ones recorded for the old experience representing that place. However, the constraints of moving through the environment are such that to pass through a place the robot can often have only a limited range of orientations. In practice, this mechanism limits the number of experiences representing a particular place. Experiences are connected with each other through transitions representing the estimated robot displacements between the experiences. Hence, experiences form a topological map of the real world, i.e. a graph with vertices (experiences) connected by edges (transitions), the so-called the experience map. However, the displacements associated with transitions allow to add a position attribute to the experiences in the graph. Indeed, upon creation of a new experience, its position attribute can be calculated from the previous experience's position and the displacement corresponding with the transition linking the two experiences. Adjusting the position attributes of the experiences each time a previously visited place is recognized as such, the position attributes of the experiences can be used to also form a semi-metric map of the environment [31].

## Supporting Information

Video S1 **Mapping of an office environment using BatSLAM.** This video shows the construction of a map in a normal office environment. The experience map shows the constructed map, and the current sonar data is represented in the panel “current cochleogram”. The raw odometry information is displayed as well, indicating the erroneous robot position estimation when only odometry information is used. Finally, the panel “Frame vs View Template” indicates which local view template is associated with the current robot pose.
(AVI)
